# The Barriers and Facilitators to Help-Seeking by Healthcare Professionals Experiencing Stress or Burnout

**DOI:** 10.1192/bjo.2026.11106

**Published:** 2026-06-30

**Authors:** Rhea Nazir, Miriam Stanyon, Karl Ryan, Susannah Basile, Subodh Dave

**Affiliations:** 1 Derbyshire Healthcare NHS Foundation Trust, Derby, United Kingdom; 2 Doctors in Distress, Watford, United Kingdom; 3 Royal College of Psychiatrists (RCPsych), London, United Kingdom

## Abstract

**Aims:**

Healthcare professionals (HCPs) face significant mental health challenges, yet barrierssuch as stigma, fear of professional repercussions, and systemic issues hinder help-seeking. At present, there is little evidence on how help-seeking in HCPs can be facilitated. Given the widespread prevalence and serious consequences of mental health difficulties among HCPs, this study aimed to explore the barriers and facilitators that help or hinder HCPs accessing and utilising mental health support and wellbeing provision within a mental health National Health Service (NHS) Foundation Trust. A further aim was to develop recommendations, informed by participants’ accounts and existing literature, to encourage uptake of mental health support and wellbeing provision and foster a culture of wellbeing across healthcare organisations.

**Methods:**

Three focus groups were conducted with healthcare professionals (doctors and nurses) employed by a mental health NHS trust. Participants represented a range of roles, backgrounds, and genders. Focus group transcripts were analysed using Reflexive Thematic Analysis, following a six-phase, non-linear process. Analysis identified key workplace stressors, coping strategies, and mitigating factors that shaped whether and how participants engaged with wellbeing support. Recommendations were developed from participants’ accounts and extrapolated in the context of existing literature.

**Results:**

Analysis generated three overarching thematic domains: (1) workplace stressors, (2) coping strategies, and (3) mitigating factors influencing help-seeking. Core stressors included overwhelming workloads, exposure to clinical risk and responsibility for patient safety, operational pressures, and the impact of serious incidents and investigations. Coping strategies ranged from individual approaches (e.g. hobbies, informal support) to organisational supports (e.g. supervision, flexible working, counselling). Barriers to help-seeking were primarily organisational and relational, including limited awareness andaccessibility of services, cultures emphasising resilience and self-sacrifice, and trust- and manager-related dynamics that shaped perceptions of safety, authenticity, and confidentiality. Key recommendations focused on addressing system-level stressors, strengthening wellbeing-focused managerial training, and improving the accessibility, credibility, and authenticity of wellbeing services.

**Conclusion:**

These findings demonstrate that help-seeking among HCPs is shaped less by individual reluctance and more by organisational culture, leadership practices, and system-level pressures. Addressing these factors may reduce burnout, encourage timely help-seeking, and enhance patient care. The study provides practical, transferable guidance for healthcare organisations seeking to develop effective interventions to support healthcare professionals’ wellbeing in the workplace.

